# Carcinogenicity of Poorly Soluble Low Toxicity Particles: Commentary on Epidemiology as a Risk Assessment “Reality Check”

**DOI:** 10.3389/fpubh.2022.920032

**Published:** 2022-07-12

**Authors:** Kenneth A. Mundt, Annette B. Santamaria, William J. Thompson, Christopher A. Bates, Corey Boles, G. Scott Dotson, Mei Yong

**Affiliations:** ^1^Cardno ChemRisk now Stantec, San Francisco, CA, United States; ^2^MY EpiConsulting, Düsseldorf, Germany

**Keywords:** epidemiology, PSLT particles, carcinogenicity, lung cancer, risk assessment, talc, taconite, carbon black

## Abstract

Inhaled particles that are poorly soluble or insoluble and of low toxicity (“poorly soluble low toxicity” or “PSLT” particles), can accumulate in the lung and at lung overload levels induce lung cancers in rats. The question of whether PSLT particles increase lung cancer risk in humans is complicated by large differences between rats and humans and the relatively large particle doses administered in animal studies even when compared with heavy human occupational exposures. We review the findings of epidemiological studies on occupational exposure to each of three different PSLT particles (carbon black, talc and taconite). The epidemiological evidence indicates that at even very high occupational exposure levels at which non-malignant respiratory diseases including pneumoconiosis and even talcosis are observed, lung cancer risks appear not to be elevated. Although positive human cancer risks might be predicted based on extrapolation from overload doses in rats to relevant exposures in humans, the epidemiological “reality check” based on the three examples indicates that these PSLT particles are unlikely to increase lung cancer risk in humans even at high occupational levels of exposure. Therefore, we propose that careful evaluation of the epidemiological evidence can serve as a “reality check” for human risk assessment and help balance the risk evaluation process.

## Introduction

Several inhaled particulate substances have been implicated in increasing the risk of cancer, and especially lung cancers in humans. For example, certain compounds of arsenic, beryllium, cadmium, chromium and nickel–as well as asbestos and crystalline silica–have been reported in various epidemiological studies to increase lung cancer risk, especially in occupational settings where these substances were produced or used and where workplace exposures were substantial (1). All of these carcinogens are believed to be directly genotoxic and therefore the observed increased risk of lung cancer may be considered biologically plausible.

Despite the relatively clear epidemiological evidence that these genotoxic substances are human lung carcinogens, some epidemiological studies demonstrate that lung cancer risks are not increased among all groups of exposed workers, and may be greatest or even limited to those most highly exposed (2–4). This suggests that for at least some of these substances there are exposure thresholds above which risk is increased, possibly reflecting the impact of one or more protective mechanisms including DNA and/or cellular repair, clearance, dissolution, chemical reduction or transformation, etc., that reduce or eliminate the carcinogenic responses to the substances. Less obvious, however, is whether inhaled particles of low toxicity that also are poorly soluble (or insoluble) in physiological fluids and that accumulate in the lung also can increase lung cancer risk. These substances are commonly referred to as “poorly soluble low toxicity” or “PSLT” particles. One classic example is coal dust (5, 6), which clearly leads to coal workers' pneumoconiosis (sometimes called “black lung”), or the related industrial chemical, carbon black, that epidemiologically have not been associated with increased risk of lung cancer.

The epidemiological research for these examples demonstrates a wide range of lung cancer risks associated with these known and potential human carcinogens. Additionally, for those substances determined to be lung cancer hazards, epidemiologically based risk evaluation may be capable of identifying evidence of exposure thresholds or the lack of increased risk associated with lower doses or with substances that are unlikely to be carcinogenic at human relevant exposures. This paper will highlight epidemiology in providing a “reality check” regarding the role of occupational exposure to a selection of PSLT particles that have been implicated in animal studies to increase lung cancer risk.

## Definition and Short Summary of Physicochemical Properties of PSLT Particles

According to the European Centre for Ecotoxicology and Toxicology of Chemicals (ECETOC), PSLT particles are those that have dissolution half-lives measured in artificial lung fluids (interstitial fluid, artificial lysosomal fluid, and artificial alveolar fluid) longer than macrophage-mediated clearance times, e.g., titanium dioxide, carbon black, talc, and printer toner (5). These particles are considered to be chemically inert and without any known specific or inherent toxicity (i.e., lack of biochemical reaction(s) between the molecules at their surface or dissolved from their surface and the embedding lung fluid) (5, 6). These properties differentiate PSLT particles from those such as crystalline silica that exhibit significant surface-related cytotoxicity (5). Bio-solubility refers to the solubility of particles in biological systems, including *in vitro* cell systems and *in vivo* biological fluids. The bio-solubility of a substance may differ substantially from its solubility in water and in different biological fluids (5, 7). The bio-solubility of particles determines how easily particles are dissolved in intracellular or extracellular fluids and cleared into the blood or lymphatic circulation, and therefore contributes significantly to clearance rates and the potential for toxicity (7). Bio-solubility is a significant factor in determining whether particles can accumulate in the lung, and the dose at which lung overload may occur (7).

## Animal Evidence-Lung Overload

Carbon black, titanium dioxide (TiO_2_) and “talc not containing asbestiform fibers” all are described by the International Agency for Research on Cancer (IARC) in Monograph Volume 93 as having “inadequate” evidence of carcinogenicity in humans. However, IARC classified the experimental animal evidence for the carcinogenicity of carbon black and TiO_2_ as “sufficient” and “limited” for talc (8). In 2010, TiO_2_ and carbon black were classified by IARC as Group 2B “possibly carcinogenic to humans” based on inhalation studies in rats and the absence of increased lung cancer in occupational epidemiological investigations and inhaled talc not containing asbestos or asbestiform fibers was classified as Group 3 - “not classifiable as to its carcinogenicity” (8).

The proposed mode of action for the animal evidence for carbon black and TiO_2_ was impaired clearance with accumulation of particles in the lung, resulting in inflammation, cell injury, production of reactive oxygen species that eventually lead to mutations and tumors in animal models. This largely was supported by studies in rats demonstrating lung tumors only at high exposure concentrations that increased particle retention, i.e., “lung overload” (9–12). However, studies conducted with mice and hamsters exposed to PSLT particles did not report increased occurrence of lung tumors (5, 8, 13). Results from several PSLT particle inhalation studies indicate the rat lung responds differently from other small animal species, non-human primates and humans [(6, 8, 14–19) as cited by (13)]. The U.S. National Institute for Occupational Safety and Health (NIOSH) considers the key steps leading to lung tumor development in the rat as particle-induced lung inflammation, oxidative stress, lung tissue damage, and epithelial cell proliferation, (i.e., tumor formation is through a secondary genotoxic mechanism) (20). The ECETOC proposed that in particle-overload exposed rats, continuous exposures promote a scenario including enhanced transfer of particles to lymph nodes, accumulation of particles in the lung, increases in lung weight, alveolar macrophage accumulation, pulmonary inflammation, alveolar epithelial hyperplasia (proliferation) and metaplasia, fibrosis and eventually cancer. These are consistent with scientific evidence that supports a threshold model for PSLT particles causing adverse respiratory tract effects in humans (7, 16).

The relevance and applicability of the lung tumor findings in rat studies to humans exposed to PSLT particles have been questioned and are the subject of several comprehensive reviews resulting from scientific conferences, workshops and/or task forces (5–7, 13, 21–23). The development of lung tumors following overload exposures to PSLT particles may be unique to rats and when observed, occurs only under the circumstances of sustained particle overload in the lungs. In contrast, heavy particle exposures resulting lung overload appear not to elicit neoplastic responses in mice or hamsters, or in larger mammals including humans and non-human primates (6).

Adverse outcome pathway (AOP) scenarios for exposure to PSLT particles progressing to the development of lung tumors have not been identified in any species other than the rat. Warheit et al. (6) has identified factors that provide compelling evidence that the rat lung tumor response to PSLT particle overload is not relevant to lung cancer risks in humans. Specifically, the conceptual AOP model for the particle overload response in rats represents a species-specific set of pathological sequelae that is not consistent with the pulmonary effects documented in PSLT particle-exposed mice/hamsters or in particle-exposed non-human primates or coal miners. In humans and primates, a large proportion of inhaled particles that deposits in the distal lung translocates across alveolar epithelial barriers to interstitial sites. This sequestration of particles in the interstitial (humans and primates) vs. alveolar (rat) compartments most likely has an impact on the types of adverse pulmonary effects observed in humans and rats. In addition, the model for long-term particle retention in the lungs of humans developed by Gregoratto et al. (24) estimates substantially greater translocation of inhaled particles into the interstitial compartment than the International Commission on Radiological Protection (ICRP) Human Respiratory Tract Model (HRTM), which correlates well with the findings of pulmonary responses reported in studies on particle-exposed monkeys and post-mortem evaluations of particle kinetics/responses in the lungs of long-term coal miners. Furthermore, there are differences in the morphology, anatomical locations, and characterization of lung tumor types in PSLT particle-overload exposed rats vs. human lung tumors e.g., those associated with cigarette smoking.

## Epidemiology as a “Reality Check”: Lung Cancer and Occupational Exposure to Carbon Black, Talc, or Taconite

IARC (8) acknowledged that some of the mechanistic steps have been shown to occur in humans exposed to poorly soluble particles (PSP), however, the group concluded that “it is not known to what extent humans are susceptible to particle-induced lung cancers” associated with PSLT particles such as carbon black and talc. While the use of data from animal studies is an important piece of evidence in the classifications of chemicals, epidemiology seems better suited for providing a “reality check” for the potential impact of exposure to PSLT particles. The clear advantage for risk evaluation and quantitative risk analysis is that epidemiological evidence directly reflects relationships between human-relevant exposures (including extreme ones historically occurring in some workplaces) and actual increased risk, if any, of human lung cancer.

### Occupational Carbon Black Exposure and Lung Cancer Risk

Carbon black is a powdered form of elemental carbon occurring as near-spherical colloidal particles and coalesced particle aggregates of colloidal size, produced by the controlled combustion or thermal decomposition of hydrocarbons. Carbon black is used in many commercial products such as rubber, particularly in tires, and in plastics (25).

Three large cohort studies of carbon black production workers investigated the association between carbon black exposure and lung cancer mortality risk. Primary results from these lung cancer studies are summarized in Table 1. A cohort mortality study of 6,634 US carbon black workers from 18 facilities with high tracing (98.5%) of vital status and over 8,000 time-weighted average measurements summarized using a job-exposure matrix (JEM) analyzed lung cancer mortality by years of employment, years since first exposure, and years since cessation of exposure. The study reported no increase in lung cancer mortality and no relationship with any exposure metric (26).

**Table 1:** Lung cancer results from cohort studies of carbon black workers.

**Reference**	**Cohort size**	**Lung cancers (*N*)**	**Period, Location** **(Reference rate)**	Lung cancer SMR*(95% CI)
Dell et al. (26)	6,634 including inception cohort 4,882	184	1940–2011 United States (national)	0.77 (0.67–0.89)
Sorahan et al. (27)	1,147	61	1951–1996 UK (England and Wales)	1.61 (1.29–2.00)
Wellmann et al. (28)	1,522	50	1976–1998 Germany (West Germany)	2.18 (1.61–2.87)
Morfeld et al. (29)**	1,528 including inception cohort 1,271	47	(West Germany) (Northe-Rhine Westphalia) (Cologne)	1.33*** (0.98–1.77) 1.27*** (0.93–1.69) 1.20*** (0.88–1.59)

*SMR, standardized mortality ratio; **more detailed analyses of data from Wellman et al. (29); ***using local reference rates and adjusted for smoking and prior exposure.

The German cohort was defined in Wellmann et al. (28), Morfeld et al. (29, 30), and involved 1,522 production workers with a cumulative follow-up from 1976 through 1998. Using national reference rates, an initial evaluation indicated an elevated standardized mortality ratio (SMR) for lung cancer, based on 50 cases (28).

Since the smoking prevalence of this cohort was particularly high, which may have resulted in an overestimation of the SMR for lung cancer, further analyses were performed to clarify the potential role of smoking and the use different (i.e., more local) referent rates. A nested case-control study also was conducted to estimate the potential effects of selection bias and confounding due to smoking, prior exposures, and being a prisoner of war during World War II. The association with carbon black exposure was no longer apparent in analyses of 50 lung cancer cases after taking into account smoking history and using regional reference rates (31). Specifically, the SMRs for lung cancer was reduced to 1.33 (95% CI: 0.98 to 1.77) and no longer statistically significantly elevated when accounting for smoking and based on the referent rates of West Germany. The SMRs for lung cancer further were reduced to 1.27 (95% CI: 0.93 to 1.69) and 1.20 (95% CI: 0.88 to 1.59), using local reference rates from North-Rhine Westphalia and from Cologne, respectively (29).

The UK cohort involved 1,147 male production workers with follow-up from 1951 through 1996. An elevated SMR of 1.61 (95% CI: 1.29–2.00) was noted for lung cancer (27). In their re-analyses, the investigators reported elevated lung cancer risk in only two of the five plants. Furthermore, the investigators focused on the potential risk of lung cancer due to carbon black exposure in the most recent 15 years of employment (32); however, this “lugging” effect (i.e., discounting earlier exposures rather than discounting more recent exposures when “lagging” exposure to account for latency) was not apparent in the German cohort.

Combining the data from these three cohort studies with different exposure levels, a meta-regression reported no exposure-response relationship with lung cancer mortality for each 10 mg/m^3^-yr increase in cumulative exposure (beta value = −0.013, SE = 0.065, *p*-value = 0.8469) (33).

### Occupational Talc Exposure and Lung Cancer Risk

Talc is a naturally occurring mineral consisting of hydrated magnesium silicate. It is used in many industrial and consumer applications such as cosmetics, baby powder, pharmaceuticals, ceramics, paper, reinforced plastics, and paint (8).

Several occupational cohort studies of talc miners and millers in regions where the talc deposits do not have more than trace contamination by asbestos have been published and updated over the last four decades. Lung cancer results from each study are summarized in Table 2 including an overall SMR based on the pooled data. These include separate cohorts of 542 talc workers in the French Pyrenees followed for mortality from 1973 to 1995 and 1,070 talc workers from four sites in the Austrian Alps followed for mortality from 1945 to 1996 (36). Talcs from both locations were considered to have low quartz content (36). A cohort study in Norway followed 390 talc miners and millers for mortality from 1952 to 2011 (38, 39). Norwegian talc reportedly contains only trace amounts of quartz, tremolite and anthophyllite (38). A cohort of 427 talc miners and millers in Vermont (US) who began working as early as 1930 were followed for mortality through 2012 (35, 40). The talc was described in the original publication as “free of asbestos and of significant quantities of free silica” (40).

**Table 2:** Lung cancer results from cohort studies of miners and millers of talc reportedly not contaminated with asbestos [updated from (34)].

**Reference**	**Period, Location**	**Cohort** **size**	**Lung cancers** **(*N*)**	Lung cancer SMR* **(95% CI)**
Fordyce et al. (35)	1940–2012 Vermont	427	32	1.44 (0.98–2.03)
Wild et al. (36)	1945–1996 France	1,070	21	1.23 (0.76–1.89)
Wild et al. (36)	1973–1995 Austria	542	7	1.06 (0.43–2.19)
Ciocan et al. (37)	1946–2020 Italy	1,749	85	1.02 (0.82–1.27)
Wergeland et al. (38)	1953–2011 Norway	390	21	1.17 (0.73–1.79)
Combined		4,178	166	1.13 (0.97–1.31)**

*SMR, standardized mortality ratio; ** Pooled SMR.

Finally, perhaps the most informative study for purposes of examining risks of lung cancer among heavily talc-exposed workers is the occupational cohort study of 1,749 miners and millers of “a very pure type of talc” in the Val Chisone region in northern Italy (37, 41). These workers were enumerated and have been followed for decades until 77% were deceased (37). Early reports document enormous dust concentrations in both mines and mills, and consequently the risk of non-malignant respiratory disease was profoundly increased, especially silicosis in miners (37, 41). Nevertheless, millers were exposed to high concentrations of pulverized talc (and unlikely silica) and had a non-significant excess of pneumoconiosis (SMR = 2.63; 95% CI: 0.96–5.73, 6 observed cases) (37).

Even without consideration for smoking, a pooled-SMR of lung cancer for the five cohorts with high historical exposures to talc conducted in five different countries was 1.13 (95% CI: 0.97–1.31). Furthermore, there is no increase in pleural cancer/mesothelioma in studies of talc miners and millers in talc with no known or detectable contamination by asbestiform minerals (42). This finding is not surprising due to the lack of a credible level of biological plausibility as well as the lack of substantial exposure to amphibole asbestos associated with these talc mining and milling operations.

### Occupational Taconite Exposure and Lung Cancer Risk

Although not commonly identified as a PSLT (5-6), taconite is an iron-rich mineral with uncertain but likely low toxicity extracted as an iron ore that has been mined in the Mesabi Iron Range in Minnesota (US) since 1865. Iron oxide, however, is considered a PSLT. Hematite, a high-grade iron ore, was originally mined in this region, but after reserves were depleted in the 1950's, the mining of the lower-grade taconite began (43). Several publications on the lung cancer mortality of miners and millers exposed to taconite dusts have been generated since the 1980's, in part addressing concerns related to the elongate mineral particle (EMP) characteristics of taconite, and the hypothesis that EMPs might behave as fibers and increase the risk of mesothelioma (44–46). The most recent studies were cohort updates published in 2014-2015 and incorporating substantially larger numbers of workers than in previous reports, with totals exceeding 40,000 (47, 48). Two publications addressed lung cancer incidence among these workers (43, 48). The results of the studies of Minnesota taconite workers and lung cancer risk are presented in Table 3.

**Table 3:** Lung cancer results from studies of taconite workers.

**Reference**	**Period**	**Size**	**Lung cancers**	**Lung cancer SMR, SIR or** OR*(95% CI)
Higgins et al. (44)	1952–1976	5,751	15	0.84 (0.47–1.38)
Cooper et al. (45, 46)	1959–1988	3,444	62	0.87 (0.52–0.86)
Allen et al. (47)	1960–2010	31,067	949	1.16 (1.09–1.24)
Allen et al. (48)	1988–2010	40,720	973	1.1 (1.0–1.3)**
Allen et al. (43)	1960–2010	3,381 controls	1,706	Exposure Hematite 0.81 (0.67–0.98)
				[0.13- <0.45]† 1.0 (0.79–1.25)
				[0.45- <2.35]† 0.98 (0.77–1.24)
				[> 2.35]† 0.82 (0.57–1.19)

*SMR, standardized mortality ratio; SIR, standardized incidence ratio; OR, odds ratio. **SIR adjusted for smoking. Unadjusted SIR, 1.3 (1.2–1.4). † EMP/cm^3^-yrs; EMP, elongate mineral particles.

Higgins et al. (44) compared the lung cancer mortality rate through 1976 of 5,751 taconite workers at one mining company with Minnesota state mortality rates. Cooper et al. (45, 46) similarly compared the lung cancer mortality rate through 1988 of 3,444 taconite workers at two mining and milling operations with Minnesota state mortality rates. Allen et al. (47) expanded the lung cancer mortality evaluation to seven companies which included 31,067 taconite miners for the study period of 1960 to 2010. Lung cancer incidence was evaluated in a taconite worker cohort established by the University of Minnesota and the Iron Range Resources and Rehabilitation Board. The cohort included 40,720 workers who were alive as of 1988 and followed for cancer incidence through 2010 (48). A case-control analysis nested in this cohort was performed, including additional participants and lung cancer occurring after 1960 (43).

The six publications on taconite workers identified (including cohort expansion and updates) have resulted in only one study with a slightly increased but statistically significant overall SMR for lung cancer among taconite miners (47). After an adjustment for smoking, no increase in lung cancer incidence was observed in the largest cohort (48). In a more detailed evaluation of the nested case-control data, no association with taconite exposure indicators was identified (43). Additionally, an absence of increased risk of lung cancer and mesothelioma in the Minnesota community has been noted (49).

## Discussion

Overall, the epidemiological evidence (i.e., the most direct, relevant data on health outcomes in humans) for carbon black, talc and taconite as examples of PSLT particles and risk of lung cancer consistently fails to demonstrate elevated risks or compelling exposure–response relationships. This finding is consistent with a meta-regression analysis for titanium dioxide (another PSLT particle not reviewed herein) that reported no elevated risks for mortality due to lung cancer (50). Therefore, the epidemiological evidence for carbon black, talc, taconite and titanium dioxide, though biologically plausible in experimental animal exposure-overload settings, does not support the hypothesis that these examples of PSLT particles increase lung cancer risks at human-relevant exposure levels.

While the “reality check” of epidemiology currently does not support the hypothesis that PSLT particles cause human lung cancer–at least for the three examples presented here–several caveats should be noted.

First, the use of mortality, while a reasonable (but not ideal) surrogate for incidence for some outcomes with high case-fatality rates including lung cancer, does present challenges, especially if mortality due to more rapidly fatal non-malignant conditions occurs (such as silicosis) before cancerous tumors are identified. Also, lung cancer excesses among workers often reflect a higher prevalence of cigarette smoking, which often is not captured and controlled in mortality studies.

Second, that heavy exposure to PSLT particles may not increase the risk of cancer and lung cancer (or mesothelioma) specifically, these exposures can result in increased risk of non-malignant respiratory diseases including the pneumoconioses, one of which is talcosis. Exposure to “dusts” in the workplace–often called “nuisance dusts”–remains common. Exposure to PSLT particles occurs across many industrial sectors such as (1) mineral mining, milling, preparation, (2) welding, asphalt production and application, (3) manufacturing of textiles, glassware, roofing, pulp and paper products, etc., and (4) nanomaterial manufacturing and use of PSLT nanoparticles. Therefore, it is reasonable to conclude that PSLT particle exposures may impact millions of workers globally. Future studies on possible health effects of PSLT exposure should consider and evaluate, when possible, earlier health endpoints as well as measure disease incidence by exposure levels.

Third, most cohort mortality studies–including those summarized here–reflect exposure, often substantial, occurring several decades prior to the study publication. It is common for historically high exposures to have been reduced as hazards were identified and protections put in place over time. Nevertheless, even if those high exposure scenarios no longer exist, and workers continue to be exposed to the same substances at substantially lower levels, epidemiological studies characterizing exposure groups still can inform risk assessments. As exposure levels continue to decline, there may come a time when no increased risks can be detected, i.e., exposures fall below thresholds of increased risk. This may have been seen in recent decades for crystalline silica, where exposure controls that prevent silicosis also prevent lung cancer that may require higher exposures than can induce silicosis. However, for historical occupational exposure to PSLT particles such as carbon black, talc, and taconite even if orders of magnitude higher than commonly seen in recent decades, these appear not to increase lung cancer risk.

The mechanisms of toxicity of inhaled PSLT particles are increasingly well understood, allowing the establishment of safe exposure levels and identification and use of adequate risk management measures based on non-cancer endpoints. Many toxicological and epidemiological studies provide data that are more relevant to human exposures than rat lung tumors observed under particle overload conditions and appropriate for quantitative risk assessment informing the derivation of exposure limits that are protective against cancer and non-cancer endpoints. For example, an Expert Workshop recently evaluated whether inflammation should be used as the key endpoint in applying Benchmark Dose (BMD); No Observed Adverse Effect Level (NOAEL) or Lowest Observed Adverse Effect Level (LOAEL) approaches with adjustments for lung dose based on physiologic differences in species (e.g., lung deposition, ventilation) (13). The Expert Workshop recommended that occupational or environmental inhalation standards should aim to prevent inflammation as it precedes more serious responses (e.g., fibrosis, cancer, mortality) observed in epidemiological studies and may be detected at lower exposures.

While the current evidence based on cohort mortality studies of workers historically heavily exposed to carbon black, talc and elongate taconite particles does not support an association between exposure to these PSLT particles and the development of respiratory cancers in human populations, there is a need for more sensitive studies on the incidence of objectively measurable health endpoints such as inflammation that are indicative of increased risk of non-malignant respiratory diseases and can inform quantitative risk analyses supporting exposure regulations that protect workers.

## Conclusion

According to ECETOC (5), promulgating regulatory levels for PSLT particles based on their possible classification as inhalation carcinogens–observed only in rat cancer studies in which exposure overload may have occurred and likely are irrelevant to human health risks–is not epidemiologically supported, and may compromise the value of chemical hazard classification schemes. For human health hazard and risk assessment, it is important to understand not only the type of potential human health effects plausibly caused by PSLT particles in experimental animals, but also whether such exposures are relevant to humans and whether the mechanisms by which PSLT particles may cause cancers in rats occur in or can be extrapolated to humans (19). The Expert Workshop panel stated that, in the absence of supporting data from other species, overload-associated lung tumors in rats do not imply a human hazard, and that lung tumors observed in rats under lung particle overload conditions are not relevant to risks in humans exposed to PSLT particles at human-relevant exposure levels (13). Furthermore, the US EPA Guidelines for Carcinogen Risk Assessment (51) acknowledges, at least in principle, the criticality epidemiological evidence in the risk evaluation process: “Epidemiologic data are extremely valuable in risk assessment because they provide direct evidence on whether a substance is likely to produce cancer in humans, thereby avoiding issues such as: species-to-species inference, extrapolation to exposures relevant to people, effects of concomitant exposures due to lifestyles. Thus, epidemiologic studies typically evaluate agents under more relevant conditions.” The epidemiological “reality check” based on the three examples considered aligns with this perspective.

## Author's Note

This paper is based on a keynote address presented by KM at the Particles and Health 2021: an International Conference Addressing Issues in Science and Regulation held October 20-21, 2021, in London, England.

## Author Contributions

KM, CBa, AS, and WT substantially contributed to the conception and design of the presentation and manuscript. MY presented the Particles and Health 2021 Conference on carbon black and substantially contributed to the carbon black section. CBo, CBa, and GD substantially contributed to the toxicological sections. All authors contributed to interpreting findings, drafting, revising the manuscript, reading, and approving the final manuscript.

## Funding

KM and MY received reimbursement for travel expenses incurred in attending and presenting at the Particles and Health 2021 Conference but were not otherwise compensated. The Conference was sponsored by IOM - the Institute for Occupational Medicine, Edinburgh and also supported by the International Carbon Black Association (ICBA).

## Conflict of Interest

KM, AS, WT, CBa, CBo, and GD are employed by Cardno ChemRisk, now Stantec and MY is owner of MY EpiConsulting, consulting firms that provide scientific advice to various clients such as governments, corporations, law firms, and various scientific/professional organizations. Their contributions to the manuscript were part of their regular employment responsibilities with their respective firms. KM, AS, and WT have provided scientific evaluation and opinions on behalf of defendants in litigation primarily in which it is alleged that talc causes mesothelioma or ovarian cancer.

## Publisher's Note

All claims expressed in this article are solely those of the authors and do not necessarily represent those of their affiliated organizations, or those of the publisher, the editors and the reviewers. Any product that may be evaluated in this article, or claim that may be made by its manufacturer, is not guaranteed or endorsed by the publisher.
